# Changes in individual and contextual socio-economic level influence on reproductive behavior in Spanish women in the MCC-Spain study

**DOI:** 10.1186/s12905-020-00936-4

**Published:** 2020-04-15

**Authors:** Inés Gómez-Acebo, Trinidad Dierssen-Sotos, Camilo Palazuelos, Gemma Castaño-Vinyals, Beatriz Pérez-Gómez, Pilar Amiano, Tania Fernández-Villa, Eva Ardanaz, Claudia Suarez-Calleja, Juan Alguacil, Ana Molina-Barceló, José J. Jiménez-Moleón, Jessica Alonso Molero, Aina Roca-Barceló, María-Dolores Chirlaque, José Pedro Fernández Vázquez, Amaia Molinuevo, Nuria Aragonés, Maria Sala Serra, Gemma Binefa, Victor Moreno, Marina Pollán, Manolis Kogevinas, Javier Llorca

**Affiliations:** 1 Consortium for Biomedical Research in Epidemiology and Public Health (CIBER Epidemiología y Salud Pública, CIBERESP), Madrid, Spain; 2 Facultad de Medicina, Universidad de Cantabria, Avda. Herrera Oria s/n, 39011 Santander, Spain; 3 ISGlobal, Barcelona, Spain; 4 IMIM (Hospital del Mar Medical Research Institute), Barcelona, Spain; 5 Universitat Pompeu Fabra (UPF), Barcelona, Spain; 6 Cancer and Environmental Epidemiology Unit, National Center for Epidemiology, Carlos III Institute of Health, Madrid, Spain; 7 Cancer Epidemiology Research Group, Oncology and Hematology Area, IIS Puerta de Hierro, IDIPHIM, Madrid, Spain; 8 Public Health Division of Gipuzkoa, BioDonostia Research Institute, San Sebastian, Spain; 9 Instituto de Biomedicina (IBIOMED), Universidad de León, León, Spain; 10 Navarra Public Health Institute, Pamplona, Navarra Spain; 11 IdiSNA, Navarra Institute for Health Research, Pamplona, Spain; 12 IUOPA, Universidad de Oviedo, Oviedo, Asturias Spain; 13 Centro de Investigación en Recursos Naturales, Salud y Medio Ambiente (RENSMA), Universidad de Huelva, Huelva, Spain; 14 Cancer and Public Health Area, FISABIO – Public Health, Valencia, Spain; 15 Department of Preventive Medicine and Public Health, University of Granada, Granada, Spain; 16 Epidemiology Unit and Girona Cancer Registry, Oncology Coordination Plan, Department of Health, Government of Catalonia, Catalan Institute of Oncology, Girona, Spain; 17 MRC-PHE Centre for Environment and Health, Department of Epidemiology and Biostatistics, School of Public Health, Imperial College London, London, UK; 18 Department of Epidemiology, Regional Health Council, IMIB-Arrixaca, Murcia University, Murcia, Spain; 19 Complejo Asistencial Universitario de León, Sacyl, León, Spain; 20 BioDonostia Research Institute, San Sebastian, Spain; 21 Department of Epidemiology and Evaluation, IMIM (Hospital del Mar Medical Research Institute), Barcelona, Spain; 22 Catalan Institute of Oncology (ICO) and Bellvitge Biomedical Research Institute (IDIBELL), Hospitalet de Llobregat, Barcelona, Spain; 23 Department of Clinical Sciences, Faculty of Medicine, University of Barcelona, Barcelona, Spain

**Keywords:** Contextual socioeconomic, Educational level, Occupation, Urban vulnerability index, Pregnancies, Abortions, Breastfeeding, Hormonal therapy

## Abstract

**Background:**

The association between socioeconomic level and reproductive factors has been widely studied. For example, it is well known that women with lower socioeconomic status (SES) tend to have more children, the age at first-born being earlier. However, less is known about to what extent the great socioeconomic changes occurred in a country (Spain) could modify women reproductive factors. The main purpose of this article is to analyze the influence of individual and contextual socioeconomic levels on reproductive factors in Spanish women, and to explore whether this influence has changed over the last decades.

**Methods:**

We performed a cross-sectional design using data from 2038 women recruited as population-based controls in an MCC-Spain case-control study.

**Results:**

Higher parent’s economic level, education level, occupational level and lower urban vulnerability were associated with higher age at first delivery and lower number of pregnancies. These associations were stronger for women born after 1950: women with unfinished primary education had their first delivery 6 years before women with high education if they were born after 1950 (23.4 vs. 29.8 years) but only 3 years before if they were born before 1950 (25.7 vs. 28.0 years). For women born after 1950, the number of pregnancies dropped from 2.1 (unfinished primary school) to 1.7 (high education), whereas it remained almost unchanged in women born before 1950.

**Conclusions:**

Reproductive behavior was associated with both individual and area-level socio-economic indicators. Such association was stronger for women born after 1950 regarding age at first delivery and number of pregnancies and for women born before 1950 regarding consumption of hormonal contraceptives or postmenopausal therapy.

## Background

The association between socioeconomic level and reproductive factors has been widely studied. Several studies suggest that sociodemographic, socioeconomic characteristics and social class would be independent risk factors associated with neonatal morbidity (preterm birth) [1–4]. Women with lower socioeconomic status (SES) tend to have more children, the age at the first born being earlier [5]. Other studied aspects have been the number of abortions, performing breastfeeding [6] or having a child small for the gestational age [7, 8]. However, most studies did not differentiate between individual and contextual socioeconomic levels. The individual socioeconomic level refers to individual characteristics such as incomes, educational level or occupation, while contextual socioeconomic level (or area deprivation indices) would refer to characteristics in the area of residence, such as unemployment and illiterate rates or social facilities (i.e. hospitals / clinics, schools, employment, living environment, violence) [9]. Area-level deprivation form a composite score. The higher the deprivation index value, the greater the level of the neighborhood deprivation. Regardless of individual socioeconomic circumstances, greater area deprivation is associated with an increased risk of premature mortality and chronic disease [10–13].

However, little is known about how the socioeconomic changes occurred in a country can modify women reproductive factors. Thus, as far as we know, the association between socioeconomic status and reproductive factors has never been studied in terms of the great social changes produced since 1970 in Spain. (Those changes encompass the economic crisis that affected the Western countries "the first petrol crisis", the end of the Spanish dictatorship in 1976, the incorporation of women into the world of employment, changes in the sexual behavior of Western countries, universal coverage in public health or an increase in the tobacco epidemic.) Therefore, in the field of study of social inequalities in health, it would be pertinent to delve into the knowledge about differentiated patterns of reproductive and neonatal health associated with social class in these periods. For instance, would we expect that university women some 60 years ago (a scarce minority) differed from the less educated ones in the same way that current university women do?

The main goal in this study is to analyze the influence of both individual SES and area-level deprivation index on reproductive factors among Spanish women, and to explore whether this influence has changed in the last 50 years. For the purpose of this study, 2038 women recruited as population controls were used in a multiple case control study conducted between September 2008 and December 2013 in 12 Spanish provinces in Spain (MCC-Spain).

## Methods

### Study design and population

The MCC-Spain is a case-control study on several types of cancers, namely breast, prostate, colorectal, gastric and chronic lymphocytic leukemia, carried out in 12 Spanish provinces. Its main characteristics have been described elsewhere [14]. For the purpose of this study, only women without cancer were considered. Selection of population controls being selected from general practitioners’ representative sample of the Spanish women, given the almost universal coverage of the national health system in Spain.

All procedures performed involving human participants were in accordance with the ethical standards of the institutional and / or national research committee, and with the 1964 Helsinki Declaration and its later amendments or comparable ethical standards. The protocol of MCC-Spain was approved by each of the ethics committees of the participating institutions. The specific study reported here was approved by the Ethical Committee of Clinical Research of Asturias, Barcelona, Cantabria, Girona, Gipuzkoa, Huelva, León, Madrid, Navarra and Valencia. Informed consent was obtained from all individual participants included in the study.

Women were recruited between 2008 and 2013 (*n* = 2038); they were selected by random from the General Practitioners roasters. First, they were contacted by phone; if they agreed to participate, they would be cited for a standardized face-to-face interview with trained interviewers. The questionnaire included information about anthropometric data, smoking habits, alcohol consumption. Body Mass Index (BMI) was calculated taking into account self-reported weight and height 1 year before the interview.

### Reproductive factors

The questionnaire also incorporated information about dichotomous reproductive factors as if ever suffering an abortion or a dead newborn, ever use of hormonal contraceptives or hormone replacement therapy, ever suffering fertility problems, ever receiving treatment for fertility problems, ever delivering a preterm or post-term newborn and also includes a reproductive quantitative factor as age at menarche, age at menopause, age at first birth, number of pregnancies, average time between pregnancies, cumulative months of breastfeeding, average breastfeeding time per child, number of abortions and number of newborns alive. We exclude records of women whose age at first birth is below 18 or above 45.

### Individual socioeconomic level

In order to measure the individual socioeconomic level, three variables were recorded. Firstly, the participant’s educational level (unfinished primary, primary studies, secondary studies and high education). Secondly, the economic level of the parents (low, middle, high). Thirdly, the longest occupation of the participant (classified according to the Spanish Occupational Classification [15, 16], further grouped into three categories: low (V), medium (IIIb, IIIc, IVa, IVb) and high (IIIa, II, I). Our questionnaire used a series of comprehensive questions to collect self-reported information on the relative socioeconomic status of participants’ parents in three categories (low, middle, and high level). Detailed data on occupational history was collected through face-to face interviews performed by trained personnel. Work was assessed through lifetime occupational history consisting of all jobs held for at least 1 year and included information on age at beginning and end of the job, job title, and the main task of the job. For this article only include the longest occupation of the participant. Seventy participants did not report their occupation and their parents’ economic level; four participants did not report the economic level of their parents and 298 did not report their occupation.

### Area-level socioeconomic index

To measure the contextual socioeconomic level, we used the Urban Vulnerability Index (Socio-Economic) (UVI-SE) as published by the Spanish Ministry of Foment [17] . It combines five indicators based on the proportion of: unemployed, unemployed aged 16–29 years old, non-fixed employed, employed without qualification, and people without studies. The UVI-SE scores range from 0 (lower vulnerability) to 1 (higher vulnerability). We categorized UVI-SE by quintiles, Q1 indicating lower vulnerability and Q5, higher vulnerability. Each participant was assigned to the UVI-SE of her area of last residence.

### Statistical analysis

The association of each SE indicator with dichotomic reproductive factors was tested using logistic regression analysis including as regressors all four SE indicators (one logistic regression for each regressor) adjusting for age at enrollment and province of recruitment. Results from logistic regression are displayed as marginal probabilities of the event occurrence with 95% confidence intervals.

The association between the SE indicators and each reproductive quantitative factor was analyzed using linear regression with all four SE indicators (one regression for each SE indicator), adjusted for age and province of recruitment. The results are shown as marginal averages with 95% confidence intervals.

All analyses were conducted for the whole sample and separately for women born before and after 1950It is noteworthy that women born in that year reached sexual maturity around 1970. This period was defined in Spain by political democratization and the corresponding social and economic changes. All statistical analyses were performed using Stata/SE-16.

## Results

### Sample characteristics

Overall, 1031 women born before 1950 with an average age of 70 years and 1007 after 1950 with a mean age of 48 years were included in the analysis. Table 1 displays the main characteristics of the sample. Compared with women born before 1950, women born after 1950 had a higher proportion of overweight and obesity, which is in line with a greater consumption of energy and alcohol per day. The proportion of smoker women was also higher in this group. Regarding reproductive factors, the proportion of women who had their menarche after 12 years old, had a more advanced age for their first birth, had a smaller number of children, and shorter breastfeeding periods was higher among women born after 1950. The use of hormonal contraceptives was also higher in this group and they also reported more fertility problems.

**Table 1:** Sample description

Variable	Category	Women born before 1950	Women born after 1950	*p*-value
		(*n* = 1031)	(*n* = 1007)	
Age, mean (sd)		70.5 (6.6)	47.9 (7.3)	< 0.001
Age, median (interquartile range)		70 (65–76)	49 (43–54)	
Geographical area, n (%)	Madrid	163 (15.8)	217 (21.5)	< 0.001
	Barcelona	251 (24.3)	152 (15.1)	
	Navarra	105 (10.2)	80 (7.9)	
	Guipuzcoa	123 (11.9)	142 (14.1)	
	Leon	106 (10.3)	99 (9.8)	
	Asturias	59 (5.7)	68 (6.8)	
	Murcia	8 (0.8)	4 (0.4)	
	Huelva	33 (3.2)	46 (4.6)	
	Cantabria	75 (7.3)	113 (11.2)	
	Valencia	34 (3.3)	35 (3.5)	
	Granada	45 (4.4)	22 (2.2)	
	Gerona	29 (2.8)	29 (2.9)	
Parent’s SE level	Low	399 (38.7)	277 (27.5)	< 0.001
	Middle	521 (50.5)	674 (66.9)	
	High	53 (5.1)	40 (4.0)	
	NA	58 (5.6)	16 (1.6)	
Education level	unfinished primary	334 (32.4)	40 (4.0)	< 0.001
	primary studies	389 (37.7)	232 (23.0)	
	secondary studies	199 (19.3)	420 (41.7)	
	high education	109 (10.6)	315 (31.3)	
Occupation level	Low	153 (14.8)	112 (11.1)	< 0.001
	middle	368 (35.7)	323 (32.1)	
	high	230 (22.3)	484 (48.1)	
	NA	280 (27.2)	88 (8.7)	
Urban Vulnerability	Q1 (lower vulnerability)	197 (19.1)	202 (20.1)	< 0.001
	Q2	169 (16.4)	129 (12.8)	
	Q3	173 (16.8)	184 (18.3)	
	Q4	156 (15.1)	137 (13.6)	
	Q5(higher vulnerability)	191 (18.5)	137 (13.6)	
	NA	145 (14.1)	218 (21.6)	
Age at menarche	≤12 years	481 (48.2)	361 (35.6)	< 0.001
	> 12 years	517 (51.8)	653 (64.4)	
Age at first delivery	< 20 years	53 (6.9)	26 (3.0)	< 0.001
	20–24 years	219 (28.4)	288 (33.0)	
	25–29 years	270 (35.0)	393 (45.0)	
	30–34 years	160 (20.7)	127 (14.5)	
	≥35 years	70 (9.1)	39 (4.5)	
Abortion	No	799 (77.5)	779 (77.4)	0.94
	Yes	232 (22.5)	228 (22.6)	
Dead newborn	No	998 (96.8)	995 (98.8)	0.002
	yes	33 (3.2)	12 (1.2)	
Number of alive newborns, mean (sd)		1.5 (1.1)	2.4 (1.7)	< 0.001
Preterm newborn	No	942 (91.4)	909 (90.3)	0.39
	Yes	89 (8.6)	98 (9.7)	
Post-term newborn	No	989 (95.9)	964 (95.7)	0.825
	Yes	42 (4.1)	43 (4.3)	
Number of deliveries	nulliparous	142 (13.81)	227 (22.72)	< 0.001
	1–2	469 (45.62)	633 (63.36)	
	≥3	417 (40.56)	139 (13.91)	
Months of breastfeeding, mean (sd)		12.9 (31.5)	7.5 (16.3)	< 0.001
Menopausal status	Postmenopausal	1029 (99.8)	443 (44.0)	< 0.001
	Premenopausal	2 (0.2)	564 (56.0)	
Age at menopause	< 50 years	234 (59.2)	409 (45.7)	< 0.001
	≥50 years	161 (40.8)	485 (54.3)	
Use of hormonal contraceptives	No	720 (70.7)	327 (32.8)	< 0.001
	yes	298 (29.3)	671 (67.2)	
Use of hormone replacement therapy	No	858 (89.7)	942 (95.6)	< 0.001
	Yes	99 (10.3)	43 (4.4)	
Fertility problems	No	970 (95.4)	915 (92.0)	0.002
	Yes	47 (4.6)	80 (8.0)	
Treatment for fertility	No	23 (48.9)	26 (32.5)	0.066
	Yes	24 (51.1)	54 (67.5)	
Body Mass Index (kg/m2)	< 18.5	28 (2.9)	11 (1.2)	< 0.001
	18.5–24	561 (58.1)	340 (37.4)	
	25.0–29.9	256 (26.5)	350 (38.5)	
	> = 30	121 (12.5)	208 (22.9)	
Energy intake (kcal/day), mean (sd)		1686.0 (523.5)	1841.7 (611.6)	< 0.001
Ethanol intake (g/day), mean (sd)		57.7 (109.0)	78.2 (127.4)	< 0.001
Tobacco use	Non-smoker	805 (78.3)	431 (43.0)	< 0.001
	former smoker	138 (13.4)	243 (24.3)	
	current somoker	85 (8.3)	328 (32.7)	

### Association between SE indicators and reproductive factors

**Fig. 1:**
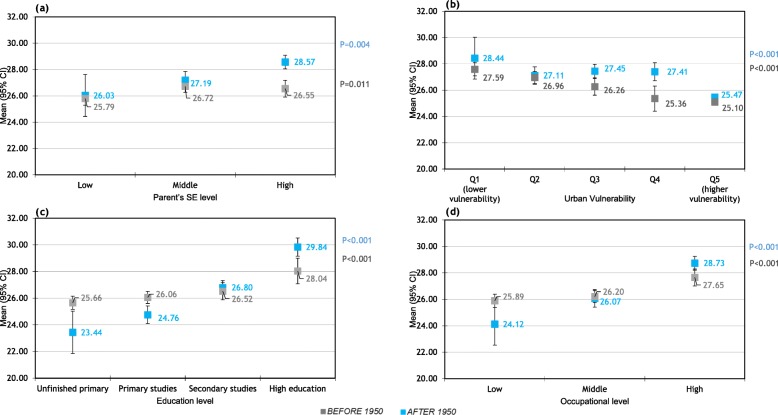
Relationship between socioeconomic level and age at first delivery.

Figure 1 shows the relationship between socioeconomic level and age at first delivery. All four SE indicators were related with age at first delivery. Higher parent’s SE, education and occupational level and lower urban vulnerability were associated with higher age at first delivery. Although these differences are seen in all women, they are higher in women born after 1950 with a difference of about 5 years between the lowest and the highest educational and occupational levels. (Averaged age at first delivery in women with unfinished primary studies equals 23.4 years vs 29.8 in women with high education; averaged age in women with low occupational level equals 24.1 vs 28.7 in women with high occupational level.) Age at first delivery was also associated with urban vulnerability, with little changes in women born before / after 1950. In this way, although in Spain, the tendency is to increase the age at first child (supplementary Figure 1), supplementary Figure 2 shows that there are changes in the tendency when stratifying by educational level: the age at first child goes down all the time in women with unfinished primary or primary study levels, while the tendency is U-shaped in women with secondary studies. Supplementary Figure 3 shows the percentages of women with university or secondary studies have largely increased in recent cohorts. Therefore, the average change in the age at first child is mostly due to changes in women’s educational level rather than changes within each educational level.

**Fig. 2:**
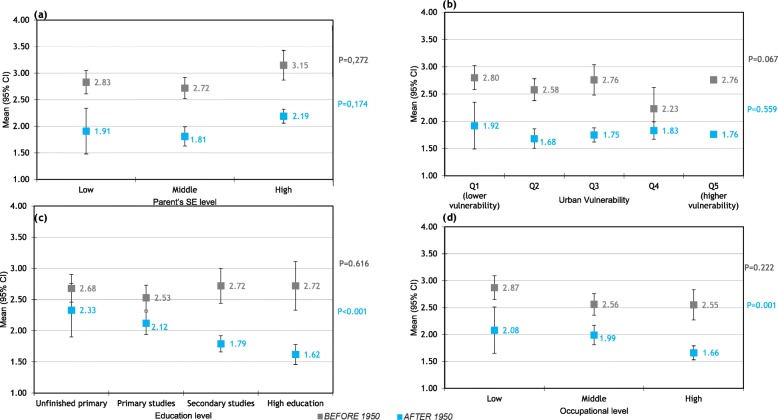
Relationship between socioeconomic level and number of pregnancies.

The association between socioeconomic level and the number of pregnancies is illustrated in Fig. 2. In women born after 1950, but not in women born before 1950, the educational and occupational levels were negatively associated with the number of pregnancies: the higher the educational and occupational level, the lower the number of pregnancies (Fig. 2c and d).

**Fig. 3:**
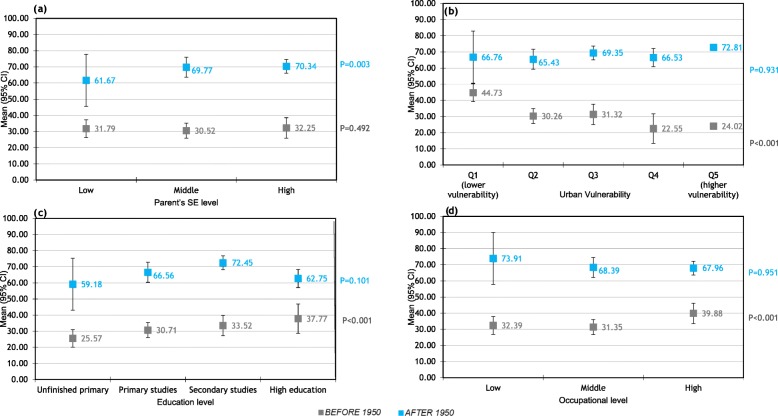
Relationship between socioeconomic level and use of hormoral contraceptives.

The percentage of women who have taken hormonal contraceptives is nearly double in women born after 1950 compared to those born before 1950 and this ratio continue almost constant across the different SE levels. The proportion of women who took the pill increased with higher educational and occupational levels and with lower urban vulnerability index in women born before 1950; halving the consumption in the lowest educational levels with respect to the highest ones (25.6% vs 37.8%). and 22.6% in urban vulnerability Q4 compared to 44.7% in Q1 (Fig. 3). In women born after 1950, however, there was no association between socioeconomic indicators and having taken hormonal contraceptives except in higher parent’s SE.

**Fig. 4:**
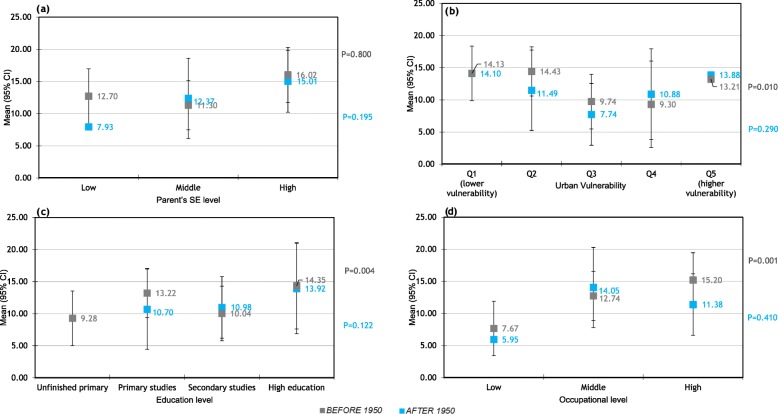
Relationship between socioeconomic level and Hormonal Replacement Therapy

Figure 4 shows the proportion of women who took hormone replacement therapy in the two periods studied. Statistical significance is only reached in the group of those born before 1950, with more educated women near doubling the use of hormone replacement therapy (HRT) of less educated women (14.4% compared to 9.2%) and women living in the less vulnerable areas doubling the use of HRT of women living in more vulnerable areas (Q1: 14.1% vs. Q4: 9.3%).

**Fig. 5:**
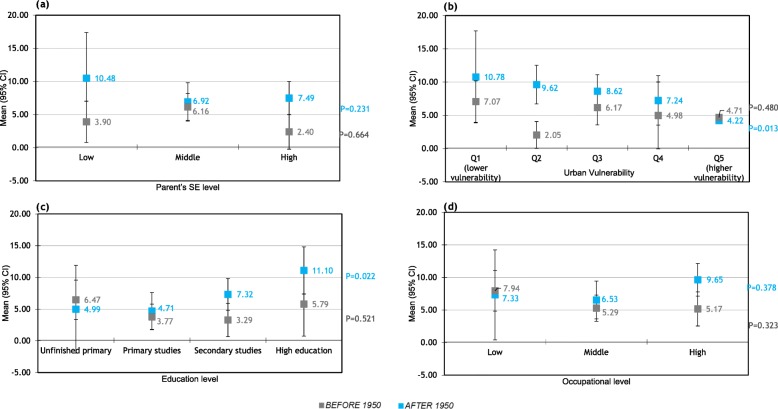
Relationship between socioeconomic level and fertility problems

Education level and urban vulnerability index were the SE indicators associated with fertility problems. Educational level displayed 6% more probability in university level for women born after 1950 compared to less educated women. Whereas, a lower urban vulnerability was associated with higher fertility problems, but only in the group of women born after 1950 (Fig. 5).

### Association between socioeconomic scores and other quantitative variables (supplementary Table 1)

#### Number of alive newborns and average time between pregnancies

The relationship between number of alive newborns and SE level mirrors that of number of pregnancies and the average time between pregnancies. In women born after 1950, education and occupational level was negatively associated with the number of pregnancies: a higher educational and occupational level was associated with fewer pregnancies.

#### Socioeconomic level and breastfeeding

The occupational level was negatively associated with months of breastfeeding (higher occupational level with less months of breastfeeding) in the whole sample and in women born before 1950. The cumulative number of months of breastfeeding was also negatively associated with education level in the whole sample. The relationship between the months of breastfeeding and SE level mirrors that of the average time of breastfeeding per child.

### Association between individual and contextual socioeconomic scores with other dichotomic variables associated with reproduction (supplementary Table 2)

#### Socioeconomic level and abortion

The percentage of women who have suffered at least an abortion was associated with higher educational and parent’s SE level in the whole sample and in women born before 1950, but all these associations disappeared in women born after 1950.

#### Socioeconomic level and dead newborn

Women with lower educational level were more prone to have a dead newborn, but this result was only reproduced in women born before 1950. No other SE indicator was associated with this event.

#### Socioeconomic level and diagnosis of fertility problems

The level of education was indicators of SE associated with fertility problems. The educational level showed a V-shaped pattern with higher probability at the university level for women born after of 1950.

#### Preterm and post-term newborn

The proportion of women with post-term newborns decreased with increasing educational level in women born after 1950. However, no SE factor was found associated with the preterm newborn.

No association could be found between SE level and age at menopause, age at menarche and average time between pregnancies.

## Discussion

The most challenging result in our study is the emerging of socioeconomic inequalities in age at first delivery, number of pregnancies, number of alive newborns, and diagnosis of fertility problems in women born after 1950. Breastfeeding, suffering abortions or dead newborns, consumption of oral contraceptives or hormonal replacement therapy were associated with socioeconomic level in women born before 1950 but not in women born after that year.

Stratifying our analysis according to being born before or after 1950 was not arbitrary. It is noteworthy that women born in that year reached their sexual maturity around 1970, which would be considered some kind of social milestone for Spanish women. Spanish women incorporated to labor market after 1970, the labor activity rate in 1976 being 55.1% for women aged 20–24 (i.e., born after 1950) and only 29.7% for women aged 25–59 (i.e., born before 1950) [18]. Secondly, sexual behavior began to change in most western countries in the 60s, involving usage of contraceptives, family planning and women taking more control of their sexual / reproductive lives. The Spanish society, however, had their sexual habits ruled by the dictatorship that ended in the 70s, including for instance late initiation of sexual relationships, one partner only, or inclusion of adultery in the penal laws until 1976 [19]. Therefore, sexual freedom and generalized accessibility to contraceptives was reached by Spanish women from 1976 on, with some delay respect to women in other western countries. Thirdly, a deep economic crisis affecting Western countries (the so-called “first oil shock”) began in 1973. This eventually led to high unemployment rates and to deep falls in birth rates, which were more intense in Spain: the average number of children per women fell from 2.90 in 1970 to 2.22 in 1980 and 1.36 in 1990 (for comparison, figures in the UK were 2.43 in 1970 and 1.83 in 1990). In the same 20-year period, the birth rate fell in Spain from 19.5 in 1970 to 15.3 in 1980 and 10.3 newborns per 1000 inhabitants in 1990 (in the UK: 16.2 in 1970 and 13.9 in 1990) [20]. Fourthly, the public health service increased its coverage from 1970 to 1980, reaching universal coverage in 1988 [21]. Finally, the continued social trend toward reduced family size instead of that in the past, in rural areas above all, where children were needed and deemed to be suitable for farming or helping at home [22].

That social context could easily explain changes when the socioeconomic inequalities decreased in women born after 1950. For instance, hormonal contraceptives were marketed in Spain from 1964 on, but their indications initially included keeping the ovary in rest, controlling menstrual cycle, and treating dysmenorrhea and acne, while their contraceptive effect was considered as an undesirable side effect [23]. For years, they were more accessible for highly educated women living in urban areas [24]. When the public health service included contraception in its portfolio, women could access it without socioeconomic differences, leading to the results we have described for women born after 1950. Hormonal replacement therapy appeared much later than hormonal contraceptives, but its usage was amplified via private practicing doctors. This eventually resulted in higher consumption for women in higher socioeconomic levels; in 2001, when most women born after 1950 had not reached menopause, the Women’s Health Initiative study found an association between hormonal replacement therapy and several cancers [25] and other chronic diseases [26], leading to a dramatic decrease in hormone replacement therapy in Spain [27].

The emergence of new socioeconomic disparities in age at first delivery and number of pregnancies in women born after 1950, however, is challenging. These inequalities appeared or were intensified in an era of universal coverage of the public health service, with free access to contraceptive methods and widely available information about them. In this regard, it is noteworthy that universal health coverage does not imply -by itself- equity in health assistance. For instance, the OECD have noticed that despite the fact that most OECD countries have achieved universal health coverage, people from the most socially disadvantaged groups tend to have worse access to health services. Possible reasons include lack of awareness of health services, poorer quality of care and co-payments for care [28–30]. In this regard, Spanish women aged 25–44 (most of their fertile age) declared having unmet needs for health care in higher percentages if their income are in the lower quintile, although the gradient associated with income level was rather mild (5.84, 3.13, 4.15, 3.52 and 3.11% for Q1 to Q5 in 2001, [31]. Admittedly, these data on unmet needs in 2001 are too late for explaining our results, but National Health Surveys carried out before 2001 did not include any question on unmet needs. Apart from this explanation, we can only speculate on whether cultural issues associated with lower both socioeconomic and educational levels or differential access to work market could have prevented women in such levels to decrease their fertility as women in higher levels did. For instance, it could be possible that women in higher socioeconomic level incorporated earlier to the work market and, thus, delayed their decisions on having their first child. It is noteworthy, in this regard, that socioeconomic-level associated inequalities were mainly in drugs usage (hormonal contraceptives and hormone replacement therapy) in women born before 1950, which could be associated with inequities in accessing health care. The main inequalities in women belonging to later generations, however, seem to be associated with their own decisions (number and age of pregnancies), not with access to medical care. Along the same lines, a recent review carried out in 2019 showed how social determinants play an important role in the stage of breast cancer in diagnosis and survival [32].

Selection of population controls is a main strength of this study. Women aged 20–85 years were enrolled in 12 Spanish provinces after being selected from general practitioners’ roasters; they can provide a representative sample of the Spanish women, given the almost universal coverage of the national health system in Spain.

Some limitations of the study should also be noted. Firstly, reproductive variables were self-reported, which could lead to recall bias; however, as women were not aware of the hypotheses of this study, we would expect that recall bias -if exists- could be non-differential. Secondly, one of the SE indicators we have use -the Urban Vulnerability Index- is ecological by nature, which makes it possible the occurrence of ecological bias. In this regard, aggregate deprivation indexes have been found good proxies of individual income but less efficient to measure education or occupational category [33].

## Conclusions

The way socioeconomic level influences reproductive behavior in Spanish women have changed throughout the time: socioeconomic inequalities in usage of hormonal contraceptives and hormone replacement therapy have disappeared in recent generations, while inequalities in number of children and age at first birth have arisen. Further research is needed to clarify whether this tendency continues in more recent generations of Spanish women.

## Supplementary information

**Additional file 1 Supplementary Figure 1.** Age at first birth without stratifying by education level. **Supplementary Figure 2.** Age at first newborn by birth cohort obtained via regression spline. **Supplementary Figure 3.** Percentage of women with each education level, according to birth cohorts. **Supplementary Table 1.** Association between socioeconomic scores and quantitative variables. Marginal averages with 95% confidence intervals. **Supplementary Table 2.** Association between individual and contextual socioeconomic scores with dichotomic variables associated with reproduction. Marginal probabilities of the event occurrence with 95% confidence intervals.

## Data Availability

All data generated or analysed during this study are included in this published article [and its additional files]. Permission to use the study database will be granted to researchers outside the study group after revision and approval of each request by the Steering Committee.

## Abbreviations

SES: Socioeconomic status
BMI: Body mass index
UVI-SE: Urban vulnerability index- socio-economic
HRT: Hormone replacement therapy

## References

[1] Villalbí JR, Salvador J, Cano-Serral G, Rodríguez-Sanz MC, Borrell C (2007). Maternal smoking, social class and outcomes of pregnancy. Paediatr Perinat Epidemiol.

[2] Garcia-Subirats I, Pérez G, Rodríguez-Sanz M, Salvador J, Jané M (2011). Recent immigration and adverse pregnancy outcomes in an urban setting in Spain. Matern Child Health J.

[3] Sparks PJ (2009). Do biological, sociodemographic, and behavioral characteristics explain racial/ethnic disparities in preterm births?. Soc Sci Med.

[4] Paul K, Boutain D, Manhart L, Hitti J (2008). Racial disparity in bacterial vaginosis: the role of socioeconomic status, psychosocial stress, and neighborhood characteristics, and possible implications for preterm birth. Soc Sci Med.

[5] Iseyemi A, Zhao Q, McNicholas C, Peipert JF (2017). Socioeconomic status as a risk factor for unintended pregnancy in the contraceptive CHOICE project. Obstet Gynecol.

[6] Clark A, Baker SS, McGirr K, Harris M. Breastfeeding peer support program increases breastfeeding duration rates among middle- to high-income women. Breastfeed Med. 2018;13:112–5. 10.1089/bfm.2017.0021. 10.1089/bfm.2017.0021 29240452

[7] Bushnik T, Yang S, Kaufman JS, Kramer MS, Wilkins R (2017). Socioeconomic disparities in small-for-gestational-age birth and preterm birth. Health Rep.

[8] Parker JD, Schoendorf KC, Kiely JL (1994). Associations between measures of socioeconomic status and low birth weight, small for gestational age, and premature delivery in the United States. Ann Epidemiol.

[9] Shavers VL (2007). Measurement of socioeconomic status in health disparities research. J Natl Med Assoc.

[10] Wild S, Macleod F, McKnight J, Watt G, Mackenzie C, Ford I (2008). Impact of deprivation on cardiovascular risk factors in people with diabetes: an observational study. Diabet Med J Br Diabet Assoc.

[11] Lawlor DA, Davey Smith G, Patel R, Ebrahim S (2005). Life-course socioeconomic position, area deprivation, and coronary heart disease: findings from the British Women’s heart and health study. Am J Public Health.

[12] Wight RG, Aneshensel CS, Miller-Martinez D, Botticello AL, Cummings JR, Karlamangla AS (2006). Urban neighborhood context, educational attainment, and cognitive function among older adults. Am J Epidemiol.

[13] Lang IA, Llewellyn DJ, Langa KM, Wallace RB, Huppert FA, Melzer D (2008). Neighborhood deprivation, individual socioeconomic status, and cognitive function in older people: analyses from the English longitudinal study of ageing. J Am Geriatr Soc.

[14] Castaño-Vinyals G, Aragonés N, Pérez-Gómez B, Martín V, Llorca J, Moreno V (2015). Population-based multicase-control study in common tumors in Spain (MCC-Spain): rationale and study design. Gac Sanit.

[15] Clasificación Nacional de Ocupaciones 1994 [Internet]. Inst. Nac. Estad. Natl. Stat. Inst. [cited 2017 Dec 12]. Available from: http://www.ine.es/jaxi/menu.do%3Ftype=pcaxis%26path=/t40/cno94%26file=inebase%26L=0.

[16] INEbase / Clasificaciones estadísticas /Clasificaciones nacionales /Clasificación Nacional de Ocupaciones. CNO / Últimos datos [Internet]. [cited 2018 Nov 2]. Available from: https://www.ine.es/dyngs/INEbase/es/operacion.htm?c=Estadistica_C&cid=1254736177033&menu=ultiDatos&idp=1254735976614.

[17] Atlas de las Vulnerabilidad Urbana en España - Atlas de la Vulnerabilidad Urbana - Observatorio de la Vulnerabilidad Urbana - Urbanismo y política de suelo - Arquitectura, vivienda y suelo - Áreas de actividad - Ministerio de Fomento [Internet]. [cited 2018 Nov 11]. Available from: https://www.fomento.gob.es/MFOM/LANG_CASTELLANO/DIRECCIONES_GENERALES/ARQ_VIVIENDA/SUELO_Y_POLITICAS/OBSERVATORIO/Atlas_Vulnerabilidad_Urbana/.

[18] Encuesta de la población activa en 1976 [Internet]. Inst. Nac. Estad. 1990 [cited 2017 Dec 12]. Available from: http://www.ine.es/jaxi/Tabla.htm?path=/t22/e308/meto_02/pae/px/l0/&file=01011.px&L=0.

[19] Llorca J, Prieto MD, Delgado-Rodríguez M (1999). Increase in cervical cancer mortality in Spain, 1951-1991. J Epidemiol Community Health.

[20] Health at a Glance: Europe 2014 | OECD READ edition [Internet]. OECD ILibrary. [cited 2017 Dec 12]. Available from: http://www.keepeek.com/Digital-Asset-Management/oecd/social-issues-migration-health/health-at-a-glance-europe-2014_health_glance_eur-2014-en.

[21] García-Armesto S, Begoña Abadía-Taira M, Durán A, Hernández-Quevedo C, Bernal-Delgado E (2010). Spain: health system review. Health Syst Transit.

[22] Kreyenfeld M, Konietzka D. Analyzing childlessness. In: Kreyenfeld M, Konietzka D, editors. Childlessness Eur contexts causes consequences [internet]. Cham: Springer Int Publishing; 2017 . p. 3–15.[cited 2020 Feb 21] Available from: https://doi.org/10.1007/978-3-319-44667-7_1.

[23] Salguero MTR. Fecundity regulation: a demographic study on contraception, sterilization, abortion and treatment of sterility in Spai. Universidad Autónoma de Barcelona; 2002.

[24] de Miguel JM. Sociología de la población y control de la natalidad en España. Revista Española de Investigaciones Sociológicas. 1980;10:15-48 .10.2307/40182773.

[25] Rossouw JE, Anderson GL, Prentice RL, LaCroix AZ, Kooperberg C, Stefanick ML (2002). Risks and benefits of estrogen plus progestin in healthy postmenopausal women: principal results from the Women’s health initiative randomized controlled trial. JAMA..

[26] Baladé Martínez L, Montero Corominas D (2016). Macías saint-Gerons D. [Utilization of hormone replacement therapy in Spain: Trends in the period 2000–2014]. Med Clin (Barc).

[27] Costas L, Sequera V-G, Quesada P, Altzibar JM, Lope V, Pérez-Gómez B (2015). Hormonal contraception and postmenopausal hormone therapy in Spain: time trends and patterns of use. Menopause N Y N.

[28] OECD. Fiscal Sustainability of Health Systems [Internet]. OECD Publishing; 2015 [cited 2018 Aug 20]. Available from: http://www.oecd-ilibrary.org/social-issues-migration-health/fiscal-sustainability-of-health-systems_9789264233386-en.

[29] OECD. Geographic Variations in Health Care [Internet]. OECD Publishing; 2014 [cited 2018 Aug 20]. Available from: http://www.oecd-ilibrary.org/social-issues-migration-health/geographic-variations-in-health-care_9789264216594-en.

[30] OECD. Health at a Glance 2017 [Internet]. 2017. Available from: https://www.oecd-ilibrary.org/content/publication/health_glance-2017-en.

[31] Ministerio de Sanidad, Consumo y Bienestar Social. Encuesta Nacional Ministerio de Sanidad, Consumo y Bienestar Social. Encuesta Nacional de Salud [National Health Survey] [Internet]. [cited 2018 Aug 20]. Available from: https://www.mscbs.gob.es/estadEstudios/estadisticas/encuestaNacional/encuestaNac2001/home.htm.

[32] Coughlin SS (2019). Social determinants of breast cancer risk, stage, and survival. Breast Cancer Res Treat.

[33] Bryere J, Pornet C, Copin N, Launay L, Gusto G, Grosclaude P, et al. Assessment of the ecological bias of seven aggregate social deprivation indices. BMC Public Health [Internet]. 2017 [cited 2018 Nov 6];17. Available from: https://www.ncbi.nlm.nih.gov/pmc/articles/PMC5240241/. 10.1186/s12889-016-4007-8 PMC5240241 28095815

