# Supplementary material for: Changes in individual and contextual socio-economic level influence on reproductive behavior in Spanish women in the MCC-Spain study
Source: BMC Womens Health. 2020 Apr 15;20:72. doi: 10.1186/s12905-020-00936-4 (PMC7160989; doi:10.1186/s12905-020-00936-4)
Supplement: Supplementary file 1 — Additional file 1 Supplementary Figure 1. Age at first birth without stratifying by education level. Supplementary Figure 2. Age at first newborn by birth cohort obtained via regression spline. Supplementary Figure 3. Percentage of women with each education level, according to birth cohorts. Supplementary Table 1. Association between socioeconomic scores and quantitative variables. Marginal averages with 95% confidence intervals. Supplementary Table 2. Association between individual and contextual socioeconomic scores with dichotomic variables associated with reproduction. Marginal probabilities of the event occurrence with 95% confidence intervals. [file 12905_2020_936_MOESM1_ESM.pdf]

Supplementary figure 1. Age at first birth without stratifying by education level

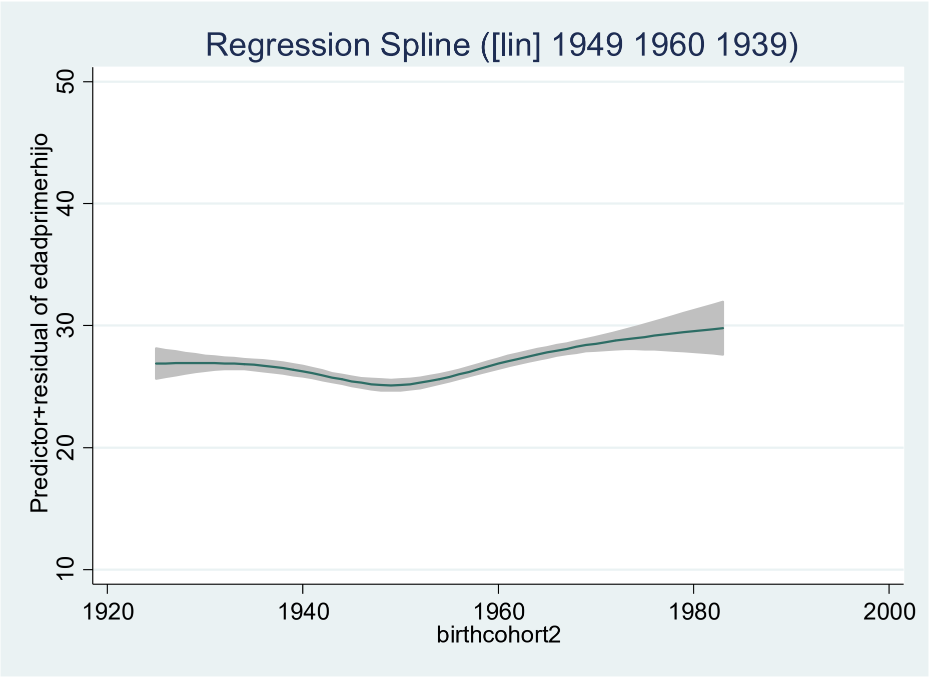

Supplementary figure 2. Age at first newborn by birth cohort obtained via regression spline

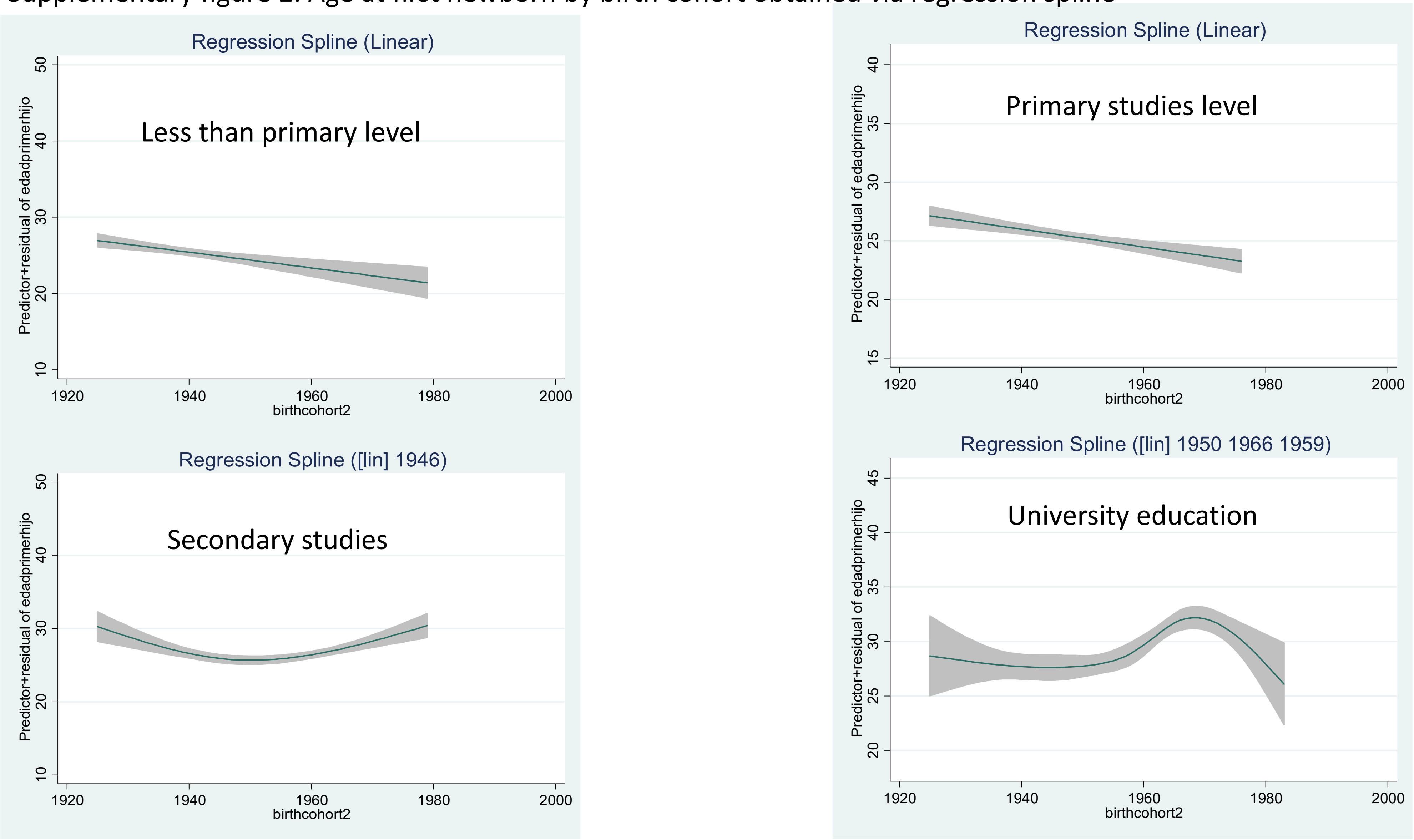

Supplementary figure 3. Percentage of women with each education level, according to birth cohorts

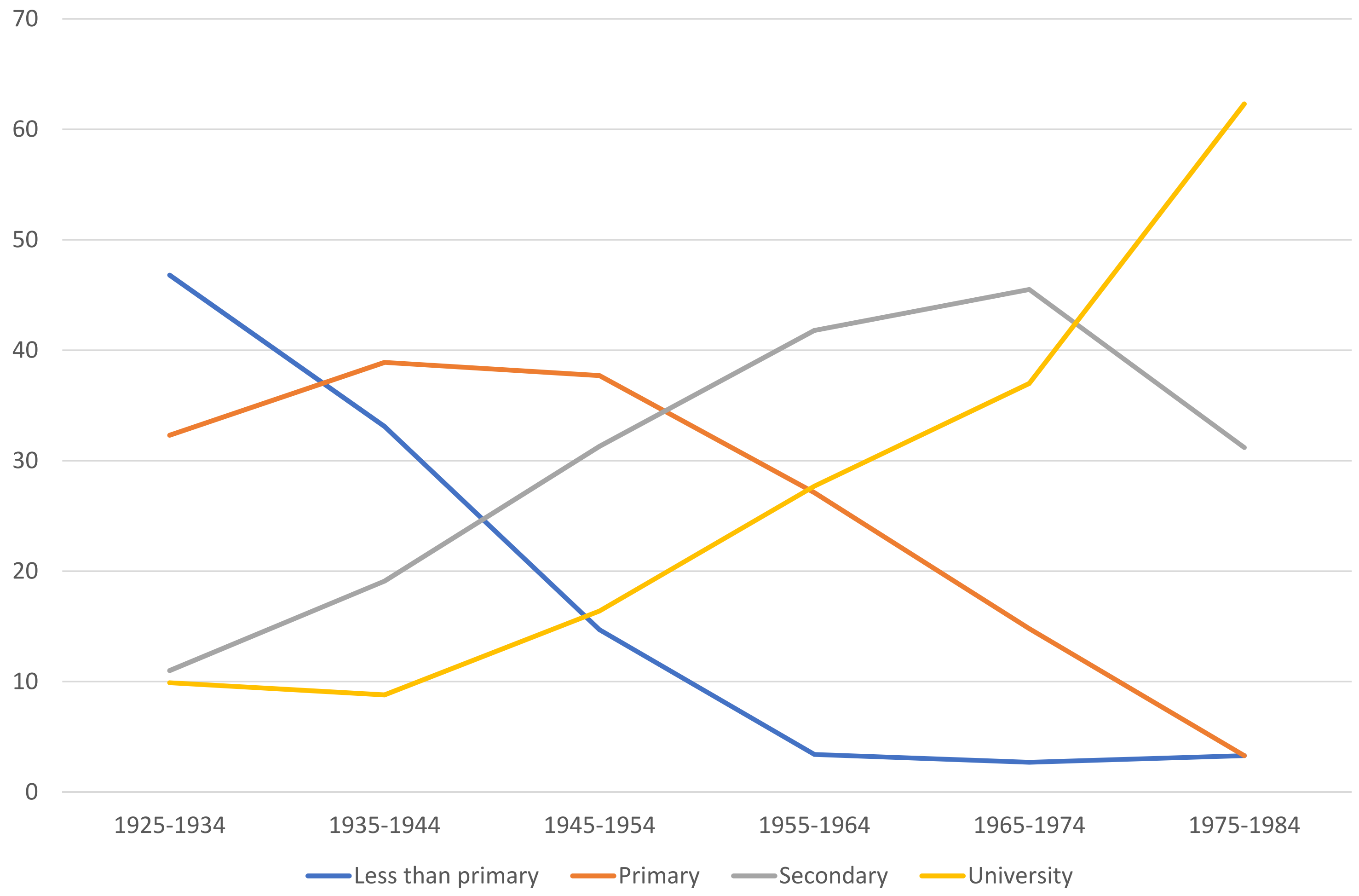

Supplementary table 1. Association between socioeconomic scores and quantitative variables. Marginal averages with 95% confidence intervals.

Reproductive variable	Socioeconomic variable	Category	Overall Mean (95% CI)	p	Women born **before** 1950 Mean (95% CI)	p	Women born **after** 1950 Mean (95% CI)	p
Age at menarche	Parent's SE level	Low	13.30 (12.63, 13.97)	0.171	13.94 (12.89, 14.99)	0.3275	12.64 (11.86, 13.42)	0.443
		Middle	13.98 (13.48, 14.47)		14.83 (13.92, 15.73)		13.19 (12.70, 13.68)	
		High	12.76 (11.00, 14.52)		13.21 (10.41, 16.01)		12.47 (10.45, 14.49)	
	Education level	Unfinished primary	13.96 (12.99, 14.92)	0.681	14.35 (13.21, 15.48)	0.5481	14.54 (12.50, 16.58)	0.352
		Primary studies	13.85 (13.16, 14.54)		14.39 (13.37, 15.41)		13.33 (12.48, 14.18)	
		Secondary studies	13.58 (12.89, 14.28)		14.72 (13.28, 16.15)		12.79 (12.18, 13.41)	
		High education	13.20 (12.33, 14.07)		13.00 (11.05, 14.95)		12.84 (12.10, 13.58)	
	Occupational level	Low	14.11 (13.06, 15.17)	0.637	15.18 (13.49, 16.87)	0.5725	13.27 (12.02, 14.53)	0.840
		Middle	13.63 (12.98, 14.27)		14.10 (13.03, 15.17)		13.16 (12.43, 13.89)	
		High	13.51 (12.87, 14.15)		14.34 (12.95, 15.72)		12.93 (12.33, 13.53)	
	Urban vulnerability	Q1 (lower vulnerability)	13.19 (12.31, 14.08)	0.701	13.28 (11.80, 14.76)	0.5806	13.11 (12.16, 14.06)	0.633
		Q2	14.12 (13.12, 15.12)		14.89 (13.32, 16.46)		13.16 (12.01, 14.30)	
		Q3	13.69 (12.79, 14.60)		14.81 (13.28, 16.33)		12.62 (11.65, 13.58)	
		Q4	13.92 (12.91, 14.93)		14.25 (12.61, 15.88)		13.63 (12.51, 14.74)	
		Q5(higher vulnerability)	13.60 (12.57, 14.63)		14.28 (12.69, 15.88)		12.54 (11.33, 13.75)	
Age at first delivery	Parent's SE level	Low	25.92 (25.53, 26.30)	<0.001	25.79 (25.34, 26.23)	0.011	26.03 (25.35, 26.70)	0.004
		Middle	26.95 (26.65, 27.25)		26.72 (26.32, 27.12)		27.19 (26.75, 27.64)	
		High	27.41 (26.39, 28.43)		26.55 (25.33, 27.77)		28.57 (26.84, 30.31)	
	Education level	Unfinished primary	25.24 (24.70, 25.78)	<0.001	25.66 (25.16, 26.16)	<0.001	23.44 (21.85, 25.03)	<0.001
		Primary studies	25.59 (25.22, 25.97)		26.06 (25.61, 26.50)		24.76 (24.11, 25.42)	
		Secondary studies	26.80 (26.39, 27.21)		26.52 (25.88, 27.16)		26.80 (26.29, 27.32)	
		High education	29.32 (28.76, 29.87)		28.04 (27.08, 28.99)		29.84 (29.17, 30.52)	
	Occupational level	Low	25.20 (24.58, 25.82)	<0.001	25.89 (25.13, 26.64)	0.001	24.12 (23.10, 25.13)	<0.001
		Middle	26.14 (25.76, 26.52)		26.20 (25.73, 26.67)		26.07 (25.47, 26.66)	
		High	28.34 (27.94, 28.75)		27.65 (26.99, 28.30)		28.73 (28.21, 29.26)	
	Urban vulnerability	Q1 (lower vulnerability)	27.99 (27.49, 28.49)	<0.001	27.59 (26.93, 28.25)	<0.001	28.44 (27.66, 29.23)	0.001
		Q2	27.05 (26.48, 27.62)		26.96 (26.26, 27.66)		27.11 (26.14, 28.08)	

		Q3	26.80 (26.29, 27.31)		26.26 (25.59, 26.92)		27.45 (26.64, 28.27)	
		Q4	26.26 (25.69, 26.83)		25.36 (24.64, 26.08)		27.41 (26.47, 28.35)	
		Q5(higher vulnerability)	25.27 (24.69, 25.84)		25.10 (24.42, 25.79)		25.47 (24.46, 26.47)	
Number of pregnancies	Parent's SE level	Low	**2.36 (2.22, 2.49)**	**0.071**	**2.83 (2.63, 3.02)**	**0.272**	**1.91 (1.75, 2.07)**	**0.174**
		Middle	**2.26 (2.17, 2.36)**		**2.72 (2.55, 2.89)**		**1.81 (1.71, 1.91)**	
		High	**2.66 (2.32, 3.01)**		**3.15 (2.62, 3.67)**		**2.19 (1.76, 2.62)**	
	Education level	Unfinished primary	2.34 (2.15, 2.54)	0.407	2.68 (2.46, 2.90)	0.616	2.33 (1.90, 2.76)	<0.001
		Primary studies	2.28 (2.15, 2.42)		2.53 (2.33, 2.73)		2.12 (1.94, 2.30)	
		Secondary studies	2.23 (2.09, 2.36)		2.72 (2.44, 3.01)		1.79 (1.66, 1.92)	
		High education	2.11 (1.94, 2.29)		2.72 (2.33, 3.10)		1.62 (1.47, 1.78)	
	Occupational level	Low	2.44 (2.24, 2.64)	0.002	2.87 (2.56, 3.17)	0.222	2.08 (1.82, 2.33)	0.001
		Middle	2.24 (2.12, 2.36)		2.56 (2.37, 2.76)		1.99 (1.84, 2.14)	
		High	2.04 (1.91, 2.16)		2.55 (2.29, 2.80)		1.66 (1.54, 1.78)	
	Urban vulnerability	Q1 (lower vulnerability)	2.39 (2.22, 2.57)	0.087	2.80 (2.51, 3.08)	0.067	1.92 (1.73, 2.10)	0.559
		Q2	2.15 (1.96, 2.35)		2.58 (2.28, 2.89)		1.68 (1.45, 1.91)	
		Q3	2.28 (2.10, 2.45)		2.76 (2.46, 3.05)		1.75 (1.56, 1.94)	
		Q4	2.04 (1.84, 2.24)		2.23 (1.92, 2.55)		1.83 (1.61, 2.05)	
		Q5(higher vulnerability)	2.29 (2.09, 2.49)		2.76 (2.45, 3.07)		1.76 (1.51, 2.00)	
Number of alive newborns	Parent's SE level	Low	2.03 (1.92, 2.14)	0.064	2.48 (2.32, 2.65)	0.317	1.59 (1.46, 1.72)	0.068
		Middle	1.91 (1.83, 1.99)		2.33 (2.19, 2.48)		1.48 (1.40, 1.56)	
		High	2.19 (1.90, 2.47)		2.58 (2.13, 3.02)		1.84 (1.51, 2.17)	
	Education level	Unfinished primary	2.19 (2.03, 2.35)	0.001	2.54 (2.35, 2.72)	0.137	2.03 (1.70, 2.36)	<0.001
		Primary studies	2.05 (1.94, 2.16)		2.34 (2.17, 2.50)		1.83 (1.69, 1.97)	
		Secondary studies	1.93 (1.82, 2.04)		2.46 (2.22, 2.69)		1.48 (1.38, 1.58)	
		High education	1.73 (1.59, 1.87)		2.12 (1.80, 2.44)		1.32 (1.20, 1.44)	
	Occupational level	Low	2.11 (1.95, 2.27)	0.000	2.52 (2.26, 2.77)	0.020	1.76 (1.56, 1.95)	0.001
		Middle	1.90 (1.80, 2.00)		2.28 (2.12, 2.44)		1.58 (1.47, 1.70)	
		High	1.68 (1.58, 1.78)		2.03 (1.82, 2.24)		1.39 (1.29, 1.48)	
	Urban vulnerability	Q1 (lower vulnerability)	2.01 (1.87, 2.15)	0.434	2.35 (2.11, 2.59)	0.480	1.60 (1.45, 1.75)	0.681
		Q2	1.88 (1.72, 2.05)		2.30 (2.04, 2.55)		1.43 (1.24, 1.61)	
		Q3	2.05 (1.90, 2.20)		2.53 (2.28, 2.77)		1.51 (1.35, 1.66)	

		Q4	1.95 (1.79, 2.11)		2.34 (2.07, 2.60)		1.52 (1.34, 1.70)	
		Q5(higher vulnerability)	2.09 (1.92, 2.26)		2.57 (2.31, 2.83)		1.55 (1.36, 1.75)	
Average time between pregnancies	Parent's SE level	Low	1.99 (1.87, 2.12)	0.025	1.88 (1.72, 2.04)	0.155	2.10 (1.90, 2.31)	0.121
		Middle	2.22 (2.12, 2.32)		2.08 (1.94, 2.23)		2.36 (2.23, 2.50)	
		High	2.06 (1.72, 2.39)		1.86 (1.42, 2.30)		2.33 (1.81, 2.85)	
	Education level	Unfinished primary	1.92 (1.73, 2.11)	0.003	1.83 (1.64, 2.02)	0.207	1.85 (1.32, 2.38)	0.012
		Primary studies	2.09 (1.95, 2.22)		2.05 (1.89, 2.22)		2.11 (1.90, 2.33)	
		Secondary studies	2.13 (1.99, 2.26)		1.99 (1.76, 2.23)		2.25 (2.09, 2.42)	
		High education	2.44 (2.25, 2.63)		2.22 (1.87, 2.56)		2.57 (2.36, 2.79)	
	Occupational level	Low	1.93 (1.73, 2.13)	0.001	1.89 (1.62, 2.16)	0.198	1.91 (1.60, 2.23)	0.002
		Middle	2.11 (1.99, 2.24)		1.99 (1.82, 2.16)		2.23 (2.05, 2.41)	
		High	2.39 (2.25, 2.52)		2.21 (1.98, 2.45)		2.52 (2.36, 2.68)	
	Urban vulnerability	Q1 (lower vulnerability)	2.19 (2.02, 2.37)	0.766	1.84 (1.60, 2.09)	0.479	2.55 (2.30, 2.80)	0.107
		Q2	2.26 (2.06, 2.46)		2.09 (1.83, 2.35)		2.51 (2.20, 2.82)	
		Q3	2.18 (2.00, 2.36)		2.07 (1.82, 2.32)		2.32 (2.06, 2.58)	
		Q4	2.15 (1.94, 2.35)		1.89 (1.60, 2.18)		2.42 (2.12, 2.72)	
		Q5(higher vulnerability)	2.06 (1.86, 2.27)		2.11 (1.84, 2.37)		1.99 (1.67, 2.31)	
Months of breastfeeding	Parent's SE level	Low	11.08 (9.11, 13.05)	0.464	14.05 (10.83, 17.28)	0.553	8.28 (6.29, 10.26)	0.672
		Middle	9.52 (8.07, 10.97)		11.69 (8.91, 14.48)		7.22 (5.98, 8.46)	
		High	9.40 (4.22, 14.58)		11.38 (2.73, 20.02)		6.93 (1.78, 12.08)	
	Education level	Unfinished primary	14.35 (11.50, 17.20)	0.019	16.22 (12.70, 19.73)	0.166	11.35 (6.14, 16.57)	0.087
		Primary studies	9.94 (7.91, 11.96)		11.07 (7.92, 14.21)		9.12 (6.95, 11.30)	
		Secondary studies	9.57 (7.52, 11.61)		11.81 (7.35, 16.27)		7.64 (6.08, 9.21)	
		High education	8.06 (5.49, 10.62)		10.74 (4.69, 16.80)		5.86 (3.97, 7.75)	
	Occupational level	Low	14.36 (11.31, 17.42)	0.001	18.91 (13.64, 24.17)	0.007	8.71 (5.64, 11.78)	0.074
		Middle	10.08 (8.22, 11.95)		11.80 (8.46, 15.13)		8.87 (7.07, 10.66)	
		High	7.14 (5.26, 9.01)		7.66 (3.33, 11.99)		6.27 (4.79, 7.75)	
	Urban vulnerability	Q1 (lower vulnerability)	7.35 (4.92, 9.78)	0.274	9.24 (4.72, 13.76)	0.402	5.17 (3.64, 6.71)	0.086
		Q2	9.59 (6.86, 12.32)		13.68 (8.90, 18.45)		4.90 (3.05, 6.76)	
		Q3	9.22 (6.74, 11.71)		10.94 (6.30, 15.58)		7.09 (5.52, 8.66)	

		Q4	11.10 (8.34, 13.87)		13.87 (8.88, 18.85)		7.92 (6.10, 9.73)	
		Q5(higher vulnerability)	11.15 (8.33, 13.98)		15.44 (10.58, 20.30)		5.73 (3.77, 7.69)	
Average time of breastfeeding	Parent's SE level	Low	4.81 (4.16, 5.47)	0.456	4.90 (4.02, 5.77)	0.756	4.81 (3.83, 5.80)	0.520
		Middle	4.90 (4.39, 5.40)		5.02 (4.24, 5.80)		4.74 (4.09, 5.38)	
		High	3.75 (2.02, 5.48)		4.07 (1.66, 6.47)		3.27 (0.74, 5.79)	
	Education level	Unfinished primary	5.32 (4.34, 6.30)	0.747	5.33 (4.30, 6.35)	0.836	5.39 (2.87, 7.92)	0.889
		Primary studies	4.72 (4.04, 5.40)		4.76 (3.85, 5.68)		4.55 (3.52, 5.57)	
		Secondary studies	4.74 (4.02, 5.46)		4.63 (3.34, 5.91)		4.84 (4.02, 5.65)	
		High education	4.60 (3.62, 5.57)		4.91 (3.02, 6.80)		4.50 (3.43, 5.56)	
	Occupational level	Low	5.81 (4.76, 6.86)	0.013	6.49 (5.04, 7.94)	0.012	4.80 (3.23, 6.37)	0.342
		Middle	5.17 (4.53, 5.82)		5.02 (4.11, 5.93)		5.31 (4.39, 6.23)	
		High	4.04 (3.34, 4.74)		3.46 (2.17, 4.74)		4.38 (3.57, 5.19)	
	Urban vulnerability	Q1 (lower vulnerability)	3.61 (2.85, 4.36)	0.234	3.81 (2.54, 5.08)	0.257	3.32 (2.55, 4.09)	0.082
		Q2	4.66 (3.78, 5.53)		5.67 (4.30, 7.03)		3.33 (2.37, 4.29)	
		Q3	4.41 (3.62, 5.19)		4.25 (2.96, 5.54)		4.55 (3.74, 5.36)	
		Q4	4.87 (3.95, 5.78)		4.97 (3.45, 6.50)		4.60 (3.67, 5.54)	
		Q5(higher vulnerability)	4.64 (3.75, 5.53)		5.46 (4.08, 6.83)		3.60 (2.61, 4.59)	
Age at menopause	Parent's SE level	Low	50.22 (49.37, 51.07)	0.284	51.15 (50.08, 52.22)	0.177	47.97 (46.67, 49.27)	0.805
		Middle	49.52 (48.81, 50.23)		50.23 (49.29, 51.17)		47.91 (46.95, 48.87)	
		High	48.51 (46.05, 50.97)		48.41 (45.38, 51.44)		49.04 (45.00, 53.08)	
	Education level	Unfinished primary	49.05 (47.87, 50.24)	0.536	49.82 (48.54, 51.10)	0.588	48.03 (44.66, 51.39)	0.971
		Primary studies	50.17 (49.28, 51.06)		51.04 (49.92, 52.15)		48.30 (46.89, 49.70)	
		Secondary studies	49.78 (48.72, 50.83)		50.57 (49.03, 52.11)		47.80 (46.63, 48.98)	
		High education	49.76 (48.36, 51.16)		50.54 (48.47, 52.62)		47.89 (46.33, 49.45)	
	Occupational level	Low	49.52 (48.17, 50.87)	0.755	50.54 (48.86, 52.22)	0.704	47.11 (44.89, 49.33)	0.141
		Middle	49.29 (48.47, 50.12)		50.08 (49.02, 51.14)		47.83 (46.62, 49.05)	
		High	49.78 (48.85, 50.71)		50.81 (49.45, 52.17)		47.89 (46.80, 48.99)	
	Urban vulnerability	Q1 (lower vulnerability)	49.47 (48.26, 50.68)	0.370	50.51 (48.93, 52.08)	0.156	47.58 (45.90, 49.25)	0.066
		Q2	49.98 (48.67, 51.29)		49.79 (48.11, 51.48)		50.20 (48.33, 52.08)	
		Q3	50.86 (49.60, 52.12)		52.16 (50.51, 53.80)		48.04 (46.29, 49.80)	

		Q4	49.04 (47.50, 50.58)	49.09 (47.19, 50.99)	48.91 (46.41, 51.41)
		Q5(higher vulnerability)	49.48 (48.09, 50.87)	50.55 (48.81, 52.29)	46.59 (44.49, 48.70)

Adjusted for age and province of recruitment

Supplementary table 2. Association between individual and contextual socioeconomic scores with dichotomic variables associated with reproduction. Marginal probabilities of the event occurrence with 95% confidence intervals.

Reproductive variable	Socioeconomic variable	Category	Overall Mean (95% CI)	p	Women born **before 1950** Mean (95% CI)	p	Women born **after 1950** Mean (95% CI)	p
Preterm newborn	Parent's SE level	Low	8.24 (6.05, 10.42)	0.235	7.16 (4.42, 9.90)	0.144	9.98 (6.34, 13.62)	0.928
		Middle	9.36 (7.61, 11.10)		9.17 (6.45, 11.89)		9.67 (7.37, 11.97)	
		High	17.45 (9.38, 25.52)		18.60 (7.74, 29.47)		15.26 (3.89, 26.64)	
	Education level	Unfinished primary	10.38 (6.46, 14.30)	0.531	8.37 (5.09, 11.65)	0.349	19.01 (4.82, 33.19)	0.502
		Primary studies	8.76 (6.34, 11.18)		8.40 (5.36, 11.44)		9.68 (5.61, 13.75)	
		Secondary studies	8.77 (6.45, 11.08)		10.44 (5.66, 15.22)		8.74 (6.01, 11.46)	
		High education	8.71 (5.79, 11.64)		4.40 (0.11, 8.69)		10.49 (6.89, 14.09)	
	Occupational level	Low	9.91 (6.03, 13.80)	0.815	10.74 (5.31, 16.17)	0.573	8.49 (3.16, 13.82)	0.928
		Middle	8.82 (6.59, 11.05)		7.46 (4.51, 10.41)		10.79 (7.28, 14.30)	
		High	9.76 (7.43, 12.09)		10.22 (5.73, 14.72)		9.64 (6.95, 12.33)	
	Urban vulnerability	Q1 (lower vulnerability)	10.77 (7.45, 14.09)	0.487	9.68 (4.76, 14.59)	0.794	12.04 (7.38, 16.69)	0.226
		Q2	7.46 (4.29, 10.63)		7.97 (3.41, 12.53)		7.21 (2.64, 11.79)	
		Q3	11.50 (7.98, 15.01)		10.39 (5.40, 15.39)		12.72 (7.64, 17.80)	
		Q4	7.11 (4.07, 10.14)		7.49 (3.17, 11.81)		6.91 (2.50, 11.32)	
		Q5(higher vulnerability)	8.52 (5.07, 11.97)		7.29 (3.14, 11.44)		10.21 (4.27, 16.15)	
post-term newborn	Parent's SE level	Low	5.62 (3.61, 7.64)	0.877	5.69 (2.81, 8.56)	0.773	6.19 (3.02, 9.36)	0.931
		Middle	4.39 (3.14, 5.65)		5.97 (3.49, 8.45)		4.18 (2.55, 5.81)	
		High	6.78 (1.10, 12.45)		2.96 (-2.66, 8.58)		11.04 (0.64, 21.45)	
	Education level	Unfinished primary	4.41 (1.31, 7.51)	0.473	4.20 (1.00, 7.41)	0.281	9.53 (-2.88, 21.94)	0.009
		Primary studies	6.94 (4.44, 9.44)		5.43 (2.68, 8.18)		10.36 (5.24, 15.47)	
		Secondary studies	5.66 (3.80, 7.53)		8.83 (4.43, 13.24)		5.43 (3.09, 7.78)	
		High education	1.52 (0.37, 2.66)		0.94 (-0.91, 2.79)		1.75 (0.34, 3.17)	
	Occupational level	Low	4.53 (1.19, 7.87)	0.417	4.70 (0.03, 9.38)	0.510	4.15 (-0.47, 8.77)	0.081
		Middle	8.60 (6.04, 11.15)		6.51 (3.32, 9.71)		10.67 (6.71, 14.62)	
		High	3.39 (1.97, 4.81)		4.88 (1.77, 7.99)		2.91 (1.28, 4.53)	
		Q1 (lower vulnerability)	5.40 (3.14, 7.65)	0.938	6.76 (2.71, 10.80)	0.438	5.15 (2.22, 8.09)	0.476

	Urban vulnerability	Q2	3.88 (1.51, 6.26)		5.27 (1.21, 9.34)		3.13 (0.09, 6.17)	
		Q3	5.16 (2.53, 7.80)		5.03 (1.29, 8.76)		5.69 (1.62, 9.75)	
		Q4	5.58 (2.50, 8.65)		4.27 (0.20, 8.34)		8.06 (2.69, 13.43)	
		Q5(higher vulnerability)	7.04 (3.47, 10.60)		6.36 (1.76, 10.97)		9.34 (2.95, 15.74)	
Abortion	Parent's SE level	Low	21.65 (18.25, 25.05)	0.023	22.53 (17.88, 27.18)	0.017	21.60 (16.46, 26.75)	0.416
		Middle	23.90 (21.39, 26.41)		24.56 (20.68, 28.43)		23.02 (19.78, 26.26)	
		High	27.75 (18.45, 37.05)		28.33 (15.83, 40.82)		27.30 (13.43, 41.18)	
	Education level	Unfinished primary	17.40 (12.84, 21.97)	0.008	17.17 (12.54, 21.81)	<0.001	24.91 (9.74, 40.08)	0.951
		Primary studies	22.57 (19.04, 26.11)		23.79 (19.20, 28.39)		22.66 (16.85, 28.48)	
		Secondary studies	22.63 (19.15, 26.11)		20.61 (14.62, 26.60)		23.09 (19.00, 27.19)	
		High education	26.33 (21.73, 30.94)		36.72 (27.21, 46.23)		21.32 (16.60, 26.05)	
	Occupational level	Low	23.59 (17.96, 29.21)	0.383	20.99 (13.74, 28.24)	0.017	26.47 (17.86, 35.09)	0.322
		Middle	22.11 (18.82, 25.40)		19.03 (14.70, 23.35)		25.46 (20.52, 30.41)	
		High	23.58 (20.31, 26.85)		30.32 (23.83, 36.81)		20.60 (16.93, 24.27)	
	Urban vulnerability	Q1 (lower vulnerability)	23.03 (18.67, 27.38)	0.014	25.52 (19.03, 32.00)	0.019	20.60 (14.84, 26.37)	0.267
		Q2	21.88 (17.03, 26.72)		24.38 (17.62, 31.15)		19.36 (12.36, 26.36)	
		Q3	21.47 (17.05, 25.88)		20.88 (14.46, 27.29)		22.60 (16.35, 28.85)	
		Q4	22.71 (17.58, 27.84)		21.73 (14.73, 28.73)		23.68 (16.19, 31.17)	
		Q5(higher vulnerability)	20.04 (14.86, 25.22)		21.44 (14.18, 28.69)		17.40 (10.19, 24.61)	
Dead newborn	Parent's SE level	Low	2.64 (1.37, 3.90)	0.416	3.13 (1.20, 5.06)	0.670	5.31 (1.17, 9.45)	0.076
		Middle	2.17 (1.27, 3.07)		4.84 (2.66, 7.02)		1.18 (0.02, 2.34)	
		High	2.08 (-0.76, 4.93)		2.27 (-2.10, 6.64)		3.66 (-3.43, 10.75)	
	Education level	Unfinished primary	4.38 (1.90, 6.87)	0.000	6.28 (2.98, 9.58)	0.033	7.80 (-3.26, 18.87)	0.162
		Primary studies	2.20 (0.94, 3.46)		2.99 (1.06, 4.92)		2.98 (-0.41, 6.38)	
		Secondary studies	1.59 (0.46, 2.71)		2.76 (0.06, 5.46)		1.89 (0.04, 3.73)	
		High education	1.12 (-0.02, 2.27)		1.20 (-1.16, 3.56)		1.77 (-0.31, 3.85)	
	Occupational level	Low	3.47 (1.17, 5.77)	0.054	5.37 (1.10, 9.64)	0.480	6.29 (-0.83, 13.41)	0.164
		Middle	1.80 (0.78, 2.83)		3.47 (1.23, 5.71)		1.98 (-0.25, 4.21)	
		High	2.07 (0.82, 3.33)		5.03 (1.26, 8.81)		1.72 (0.03, 3.41)	
	Urban vulnerability	Q1 (lower vulnerability)	1.89 (0.47, 3.32)	0.204	3.13 (0.35, 5.91)	0.215	2.29 (-0.92, 5.49)	0.903
		Q2	2.96 (1.03, 4.89)		4.44 (0.93, 7.96)		4.98 (-0.66, 10.63)	

		Q3	1.35 (0.04, 2.67)		2.19 (-0.26, 4.64)		1.93 (-1.85, 5.71)	
		Q4	2.18 (0.43, 3.93)		2.98 (0.06, 5.90)		4.23 (-1.63, 10.09)	
		Q5(higher vulnerability)	4.54 (1.75, 7.33)		8.26 (2.75, 13.78)		3.56 (-1.69, 8.80)	
Fertility problems	Parent's SE level	Low	6.71 (4.64, 8.77)	0.772	3.90 (1.89, 5.91)	0.664	10.48 (6.59, 14.37)	0.231
		Middle	6.50 (5.05, 7.94)		6.16 (3.91, 8.41)		6.92 (5.00, 8.83)	
		High	4.66 (0.21, 9.12)		2.40 (-2.22, 7.03)		7.49 (-0.60, 15.58)	
	Education level	Unfinished primary	6.76 (3.48, 10.03)	0.011	6.47 (3.35, 9.60)	0.521	4.99 (-1.92, 11.90)	0.022
		Primary studies	4.54 (2.69, 6.38)		3.77 (1.76, 5.78)		4.71 (1.81, 7.61)	
		Secondary studies	5.89 (3.99, 7.79)		3.29 (0.67, 5.91)		7.32 (4.83, 9.82)	
		High education	8.60 (5.69, 11.52)		5.79 (0.76, 10.82)		11.10 (7.40, 14.81)	
	Occupational level	Low	7.78 (4.25, 11.32)	0.544	7.94 (2.99, 12.89)	0.323	7.33 (2.42, 12.23)	0.378
		Middle	6.01 (4.10, 7.93)		5.29 (2.77, 7.81)		6.53 (3.76, 9.30)	
		High	7.75 (5.72, 9.78)		5.17 (1.95, 8.40)		9.65 (6.95, 12.35)	
	Urban vulnerability	Q1 (lower vulnerability)	9.10 (6.08, 12.11)	0.013	7.07 (3.10, 11.04)	0.480	10.78 (6.43, 15.14)	0.013
		Q2	5.32 (2.59, 8.04)		2.05 (-0.25, 4.35)		9.62 (4.25, 14.98)	
		Q3	7.48 (4.53, 10.44)		6.17 (2.27, 10.07)		8.63 (4.32, 12.94)	
		Q4	6.04 (3.23, 8.85)		4.98 (1.57, 8.39)		7.24 (2.67, 11.81)	
		Q5(higher vulnerability)	4.34 (1.88, 6.80)		4.71 (1.02, 8.40)		4.22 (0.76, 7.67)	
Treatment for fertility	Parent's SE level	Low	57.69 (42.09, 73.29)	0.095	40.49 (12.78, 68.21)	0.038	66.90 (47.92, 85.87)	0.625
		Middle	67.49 (56.41, 78.56)		71.56 (52.14, 90.98)		64.63 (50.12, 79.14)	
		High	16.38 (-15.96, 48.71)				26.74 (-23.40, 76.89)	
	Education level	Unfinished primary	51.99 (25.93, 78.05)	0.109	47.15 (20.87, 73.43)	0.568		0.658
		Primary studies	68.11 (48.45, 87.76)		62.90 (32.90, 92.90)		82.22 (60.24, 104.20)	
		Secondary studies	69.46 (53.97, 84.95)		70.92 (27.17, 114.67)		62.39 (44.16, 80.62)	
		High education	56.98 (37.88, 76.09)		69.90 (17.48, 122.32)		61.27 (43.28, 79.25)	
	Occupational level	Low	64.49 (44.18, 84.81)	0.233	64.87 (32.82, 96.92)	0.398	58.16 (23.79, 92.53)	0.765
		Middle	73.54 (60.30, 86.77)		32.10 (2.83, 61.37)		81.27 (65.62, 96.92)	
		High	56.04 (42.04, 70.03)		59.76 (6.64, 112.87)		57.12 (41.96, 72.27)	
	Urban vulnerability	Q1 (lower vulnerability)	77.48 (64.30, 90.67)	0.023	71.59 (43.29, 99.89)	0.032	75.77 (56.56, 94.99)	0.403
		Q2	61.98 (34.62, 89.34)		63.00 (5.04, 120.95)		64.89 (32.87, 96.91)	
		Q3	59.65 (39.78, 79.53)		48.95 (12.43, 85.46)		58.74 (32.41, 85.07)	

		Q4	60.26 (36.57, 83.95)		55.13 (20.16, 90.09)		66.77 (30.44, 103.11)	
		Q5(higher vulnerability)	24.02 (-2.79, 50.84)		28.64 (-18.49, 75.76)		21.08 (-17.88, 60.03)	
Oral contraceptives	Parent's SE level	Low	48.45 (44.81, 52.10)	<0.001	31.79 (27.04, 36.53)	0.492	61.67 (55.79, 67.54)	0.003
		Middle	51.01 (48.35, 53.66)		30.52 (26.64, 34.41)		69.77 (66.27, 73.28)	
		High	52.00 (42.52, 61.49)		32.25 (19.53, 44.96)		70.34 (56.17, 84.52)	
	Education level	Unfinished primary	42.08 (35.94, 48.23)	<0.001	25.57 (20.08, 31.06)	<0.001	59.18 (43.07, 75.29)	0.101
		Primary studies	49.07 (45.26, 52.88)		30.71 (26.05, 35.38)		66.56 (60.36, 72.76)	
		Secondary studies	54.43 (50.71, 58.14)		33.52 (27.20, 39.84)		72.45 (68.19, 76.72)	
		High education	48.37 (43.67, 53.07)		37.77 (28.64, 46.89)		62.75 (57.10, 68.39)	
	Occupational level	Low	55.34 (49.40, 61.28)	<0.001	32.39 (24.37, 40.40)	<0.001	73.91 (65.79, 82.04)	0.951
		Middle	52.16 (48.56, 55.75)		31.35 (26.46, 36.25)		68.39 (63.31, 73.48)	
		High	55.45 (51.96, 58.95)		39.88 (33.54, 46.21)		67.96 (63.71, 72.22)	
	Urban vulnerability	Q1 (lower vulnerability)	55.16 (50.37, 59.96)	0.001	44.73 (37.36, 52.10)	<0.001	66.76 (59.79, 73.73)	0.931
		Q2	47.90 (42.61, 53.19)		30.26 (23.23, 37.30)		65.43 (57.24, 73.62)	
		Q3	49.85 (44.92, 54.77)		31.32 (24.19, 38.45)		69.35 (62.56, 76.14)	
		Q4	43.77 (38.43, 49.12)		22.55 (16.01, 29.10)		66.53 (58.51, 74.54)	
		Q5(higher vulnerability)	46.69 (40.99, 52.39)		24.02 (17.34, 30.71)		72.81 (64.62, 81.01)	
Hormonal replacement therapy	Parent's SE level	Low	11.55 (8.34, 14.76)	0.394	12.70 (8.85, 16.54)	0.800	7.93 (2.74, 13.12)	0.195
		Middle	11.07 (8.72, 13.43)		11.30 (8.28, 14.32)		12.37 (8.28, 16.46)	
		High	14.97 (5.91, 24.03)		16.02 (5.25, 26.80)		15.01 (-3.81, 33.83)	
	Education level	Unfinished primary	7.76 (4.06, 11.47)	0.003	9.28 (5.03, 13.52)	0.004		0.122
		Primary studies	12.50 (9.24, 15.76)		13.22 (9.40, 17.03)		10.70 (4.44, 16.95)	
		Secondary studies	10.35 (7.09, 13.61)		10.04 (5.78, 14.29)		10.98 (6.17, 15.78)	
		High education	13.53 (8.72, 18.34)		14.35 (7.62, 21.07)		13.92 (6.88, 20.97)	
	Occupational level	Low	6.35 (2.28, 10.41)	0.003	7.67 (2.17, 13.17)	0.001	5.95 (-1.92, 13.82)	0.410
		Middle	12.95 (9.67, 16.22)		12.74 (8.84, 16.64)		14.05 (8.04, 20.06)	
		High	13.12 (9.61, 16.62)		15.20 (10.08, 20.32)		11.38 (6.60, 16.15)	
	Urban vulnerability	Q1 (lower vulnerability)	13.49 (9.15, 17.82)	0.006	14.13 (8.80, 19.45)	0.010	14.10 (5.93, 22.28)	0.290
		Q2	13.05 (8.52, 17.58)		14.43 (8.68, 20.18)		11.49 (3.65, 19.33)	
		Q3	7.85 (4.16, 11.55)		9.74 (4.58, 14.89)		7.74 (0.40, 15.08)	
		Q4	9.09 (4.65, 13.53)		9.30 (4.23, 14.38)		10.88 (-0.78, 22.55)	

		Q5(higher vulnerability)	12.48 (6.93, 18.04)	13.21 (6.53, 19.88)	13.88 (1.16, 26.61)

Adjusted for age and province of recruitment
